# One-year outcome of robotical vs. manual percutaneous coronary intervention

**DOI:** 10.1007/s00392-024-02524-0

**Published:** 2024-08-21

**Authors:** Constantin von zur Mühlen, Marvin Jeuck, Timo Heidt, Thomas Maulhardt, Tau Hartikainen, Alexander Supady, Ingo Hilgendorf, Dennis Wolf, Klaus Kaier, Dirk Westermann, Jonathan Rilinger

**Affiliations:** 1 Department of Cardiology and Angiology, Faculty of Medicine, Heart Center Freiburg University, University of Freiburg, Hugstetterstr. 55, 79106 Freiburg, Germany; 2 Max Grundig Klinik, Bühl, Germany; 3 Interdisciplinary Medical Intensive Care, Faculty of Medicine, Medical Center, University of Freiburg, Freiburg, Germany; 4 Heidelberg Institute of Global Health, University of Heidelberg, Heidelberg, Germany; 5 Institute of Medical Biometry and Statistics, Faculty of Medicine, University Medical Center Freiburg, University of Freiburg, Freiburg, Germany

**Keywords:** Robotic, R-PCI, Percutaneous coronary intervention, Coronary artery disease, Outcome

## Abstract

**Background:**

Robotic-assisted percutaneous coronary intervention (R-PCI) is a promising technology for optimizing the treatment of patients with coronary heart disease. For a better understanding of the potential of R-PCI in clinical routine compared to conventional manual PCI (M-PCI) both initial treatment success of the index procedure and long-term outcome have to be analysed.

**Methods:**

Prospective evaluation from the FRiK (DRKS00023868) registry of all R-PCI cases with the CorPath GRX Cardiology by Siemens Healthineers and Corindus in the Freiburg University Heart Center between 04/2022 and 03/2023. Index procedure success and safety, radiation dose of patients and personnel, and 1-year outcome will be reported. Findings will be compared to a prospective control group of M-PCI patients treated by the same team of interventionalists during the same observation period.

**Results:**

Seventy patients received R-PCI and were included in the registry. PCI success rate was 100%, with 19% requiring manual assistance. No complications (MACE—major adverse cardiovascular events) occurred. Compared with 70 matched-pair M-PCI patients, there was a higher median procedural time (103 min vs. 67 min, *p* < 0.001) and fluoroscopy time (18 min vs. 15 min, *p* = 0.002), and more contrast volume was used (180 ml vs. 160 ml, *p* = 0.041) in R-PCI vs. M-PCI patients. However, there was no significant difference of the dose-area product (4062 vs. 3242 cGycm^2^, *p* = 0.361). One year after the intervention, there was no difference in mortality, rehospitalisation, unscheduled PCI or target vessel failure. Health-related quality of life evaluation 6 and 12 months after the index procedure (NYHA, CCS, SAQ7 and EQ-5D-5L) was similar in both groups.

**Conclusion:**

R-PCI is feasible and safe. Compared to M-PCI, index procedure success rate is high, safety profile is favourable, and manual assistance was required in only few cases. At 1-year follow-up results for R-PCI vs. M-PCI considering mortality, rehospitalisation, morbidity and target vessel failure were equal.

**Supplementary Information:**

The online version contains supplementary material available at 10.1007/s00392-024-02524-0.

## Background

Robotics in interventional cardiology (R-PCI) is an innovative technology that enables coronary intervention being controlled using joysticks and a control console [1]. The R-PCI setup consists of a robotic arm, which is attached to the catheter laboratory table and connected to the patient via the inserted guiding catheter. The robotic arm is controlled via a control console, which is located separately from the patient, either behind a radiation shield or in a remote room. Previous studies have shown that R-PCI can be performed safely [2, 3] and that R-PCI delivers comparable periinterventional results to conventional, manual PCI (M-PCI) [4–7]. Moreover, radiation exposure and musculoskeletal injuries by radiation protection [8, 9] to the personnel may be reduced or avoided by R-PCI [10].

Nevertheless, to date, there is limited data on the intermediate and long-term outcome of R-PCI. Therefore, aim of this study was to investigate the hypothesis that R-PCI can achieve comparable long-term results to M-PCI. For this purpose, we conducted a prospective study of R-PCI patients while also establishing a prospective control group of M-PCI patients, which were treated by the same team of interventionalists during the same period of time with a 1-year follow-up.

## Methods

This study followed the REporting of studies Conducted using Observational Routinely-collected health Data (RECORD) reporting guidelines [11].

### Study population

Between April 2021 and March 2023, data of all R-PCI procedures at the University Heart Center Freiburg—Bad Krozingen, Germany, were collected prospectively. Prospective, blinded data collection and analysis for this study was approved by the local ethics committee (EK-Freiburg 20-1344). All patients signed written informed consent. The study was registered with DRKS (DRKS00023868).

All patients who underwent diagnostic coronary angiography at our center during this period were evaluated for R-PCI. Patients with ST-ACS were not treated with R-PCI to avoid delaying recanalization due to the preparatory steps of R-PCI. Patients with unstable angina and NSTE-ACS with low enzyme dynamics and cardiorespiratory stability were also evaluated for R-PCI. Only patients with lesions considered suitable for R-PCI as assessed by the responsible physician were treated robotically. Chronic total occlusions as well as bifurcation lesions were excluded. All patients were followed up prospectively for 1 year. Clinical data was collected on the procedures and clinical events (endpoints) as well as HRQL after both 6 and 12 months after index-PCI by telephone interview.

### Robotic-assisted percutaneous coronary intervention (R-PCI) and manual PCI (M-PCI)

R-PCI was performed using the CorPath GRX Cardiology by Siemens Healthineers & Corindus. In addition to the prospective R-PCI cohort, an additional prospective M-PCI cohort was established at the same time period. M-PCI procedures were performed by the same team of interventionalist in the same period of time, to achieve an equal skill level between the R-PCI and M-PCI procedures. R-PCI was performed by a team of three experienced interventionalists, with one performing the PCI at the control console in the control room and one of the others executing the wire and catheter changes on the robotic arm in the catheter laboratory (for the description of the processes of the R-PCI vs. M-PCI see supplemental table S1).

### Endpoints

#### Primary endpoints

Evaluation of R-PCI therapy success in terms of the rate of successful PCI (%, residual diameter stenosis < 20% and TIMI III), the need for manual assistance (%) and the safety of R-PCI defined as major adverse cardiovascular events (%, MACE). MACE was defined as: Death from any cause, cerebrovascular event (stroke), documented myocardial infarction (defined according to the Fourth Universal Definition of Myocardial Infarction (2018) of the European Society of Cardiology) [12], repeat revascularisation (PCI and/or coronary artery bypass grafting (CABG)).

#### Secondary endpoints

Secondary endpoints were procedural characteristics of R-PCI like intervention time (min), fluoroscopy time (min), contrast volume used (ml) as well as radiation dose for the patient (Dose-area product—cGycm^2^) and the physician (µSv). Moreover, six- and twelve-month follow-up regarding all-cause mortality (%), rehospitalization rate (%), rate of unscheduled PCI and target vessel failure (%, cardiac death, target vessel myocardial infarction [12] or ischaemia-related target vessel revascularization) as well as health-related quality of life (NYHA score [13], Canadian Cardiovascular Society (CCS) Angina Grade [14], EuroQoL 5D (EQ-5D-5L) [15] and Seattle angina questionnaire—7 short form (SAQ-7) [16] was analyzed.

The qualitative description of the coronary lesions was based on the Modified ACC/AHA Lesion-Specific-Classification [17] and the SYNTAX I score [18].

### Statistical analysis

Continuous variables are presented as median (25–75th percentile), categorical variables as numbers and percentages. Mann–Whitney *U* test was used for analysis of continuous variables, Fisher’s exact test for categorical variables. Survival curves were calculated using the Kaplan–Meier method. In order to reduce confounders in patient selection between the R-PCI and M-PCI groups, a propensity score matched pair analysis (nearest neighbour matching, caliper 0.1) was performed using “Reason for PCI, Modified ACC/AHA Lesion-Specific Classification of primary lesion and SYNTAX I score” as matching parameters. Statistical calculations were performed using IBM SPSS statistics 25.0 (Armonk, NY: IBM Corp, 2017) and Stata 17.0 (StataCorp. 2021. Stata Statistical Software: Release 17. College Station, TX: StataCorp LLC). Figures were produced using Stata 17.0 (StataCorp. 2021. Stata Statistical Software: Release 17. College Station, TX: StataCorp LLC) and GraphPad Prism (V9., GraphPad Software, San Diego, California USA).

## Results

### Patients

A total of 70 patients were treated with R-PCI during the study period from 04/2022 to 03/2023 (total PCIs in this period 1297, R-PCI rate 5.3%). 149 patients with M-PCI were prospectively enrolled as a control group during the same time period. Of the 149 patients with M-PCI, 70 were selected using the matched pair process and compared with the R-PCI patients.

### Periprocedural outcome of R-PCI

In 70 R-PCIs performed, the success rate for PCI was 100%. The rate of manual assistance was 18.6%. Periprocedural complications defined as MACE were not observed in any patient (0%). The radiation dose for the physician in the catheter laboratory supporting the robotic arm during the interventions was 0.6 (0.2–1.0) µSv. The physician at the control console did not receive any intervention-dependent radiation dose. Pre-programmed algorithms that were used for the R-PCIs were Spin 27 (38.6%), Dotter 20 (28.6%), Retreat on retract 12 (17.1%) and Wiggle 3 (4.3%). R-PCI was combined with advanced coronary diagnostics and therapy with iFR—SyncVision use in 17 (24.3%) patients, OCT in 10 (14.3%) patients and IVL in 2 (2.9%) patients.

### Baseline characteristics of matched pair cohorts

Baseline characteristics were well matched between the groups (supplemental figure S1) with no significant differences in sex distribution, the prevalence of cardiovascular risk factors like hypertension, diabetes mellitus, hyperlipidemia, previous percutaneous coronary intervention (PCI), and congestive heart failure (Table 1). However, there were notable differences in age, with the M-PCI group being significantly older (median age: 77 vs. 69.5 years, *p* = 0.016) and having a higher body mass index (BMI) (25.9 kg/m^2^ vs. 27.9 kg/m^2^, *p* = 0.044). Additionally, the smoking rate was significantly higher in the R-PCI group (45.7% vs. 27.1%, *p* = 0.035). Importantly, there were no significant differences of the matching parameters indication for PCI, Modified ACC/AHA Lesion-specific classification and SYNTAX score of the primary target stenosis. Except for a slightly higher initial CK before the intervention in the R-PCI group, there was no relevant difference in the lab values (supplemental table S2). In the M-PCI group more true bifurcation lesions were treated (8 (11.4%) vs. 0 (0.0%), *p* = 0.006) and degree of primary lesion stenosis was slightly higher (90.0 (80.0–99.0) vs. 90.0 (80.0–90.0), *p* = 0.048, supplemental table S3).

**Table 1:** Baseline characteristics

	M-PCI (*n* = 70)	R-PCI (*n* = 70)	*p* value
Age (years)	77.0 (68.0–82.0)	69.5 (63.0–80.0)	**0.016**
Sex (male)	56 (80.0%)	54 (77.1%)	0.837
BMI (kg/m^2^)	25.9 (24.1–30.1)	27.9 (24.7–31.2)	**0.044**
Cardiovascular risk factors			
Hypertension	51 (72.9%)	50 (71.4%)	1.000
Diabetes mellitus	25 (35.7%)	30 (42.9%)	0.489
Hyperlipidemia/dyslipidemia	36 (51.4%)	35 (50.0%)	1.000
Smoking	19 (27.1%)	32 (45.7%)	**0.035**
Family history of CAD	10 (14.3%)	14 (20.0%)	0.502
CAD	44 (62.9%)	42 (60.0%)	0.862
Previous PCI	43 (61.4%)	42 (60.0%)	1.000
Previous CABG surgery	5 (7.1%)	2 (2.9%)	0.441
Previous MI	27 (38.6%)	18 (25.7%)	0.147
Congestive heart failure	10 (14.3%)	11 (15.7%)	1.000
LVEF (%)	55.0 (45.0–55.0)	55.0 (45.0–55.0)	0.540
Chronic renal failure	14 (20.0%)	8 (11.4%)	0.245
NYHA score baseline	2.0 (1.0–3.0)	2.0 (1.0–3.0)	0.351
CCS angina scale baseline	3.0 (0.0–4.0)	1.5 (0.0–4.0)	0.328
Indication for PCI			
STE-ACS	3 (4.3%)	0 (0%)	0.962
NSTE-ACS	3 (4.3%)	6 (8.6%)	
Unstable angina pectoris	23 (32.9%)	25 (35.7%)	
Stable angina pectoris	41 (58.5%)	39 (55.7%)	
Modified ACC/AHA lesion-specific classification of the primary target stenosis type			
Type A	12 (17.1%)	17 (24.3%)	0.503
Type B	40 (57.1%)	34 (48.6%)	
Type C	18 (25.7%)	19 (27.1%)	
SYNTAX score of the primary target stenosis	5.0 (3.0–7.0)	6.0 (4.0–8.0)	0.185

*ACC/AHA* American College of Cardiology/Amercian Heart Association, *BMI* body mass index, *CABG* coronary artery bypass grafting, *CAD* coronary artery disease, *CCS* Canadian Cardiovascular Society, *LVEF* left ventricular ejection fraction, *MI* myocardial infarction, *M-PCI* manual PCI, *NSTE-ACS* non-ST-segment elevation acute coronary syndrome, *NYHA* New York Heart Association, *PCI* percutaneous coronary intervention, *R-PCI* robotic-assisted PCI, *STE-ACS* ST-segment elevation acute coronary syndrome, *SYNTAX* synergy between PCI with taxus and cardiac surgery

### Periprocedural outcomes of matched pair cohorts

**Fig. 1:**
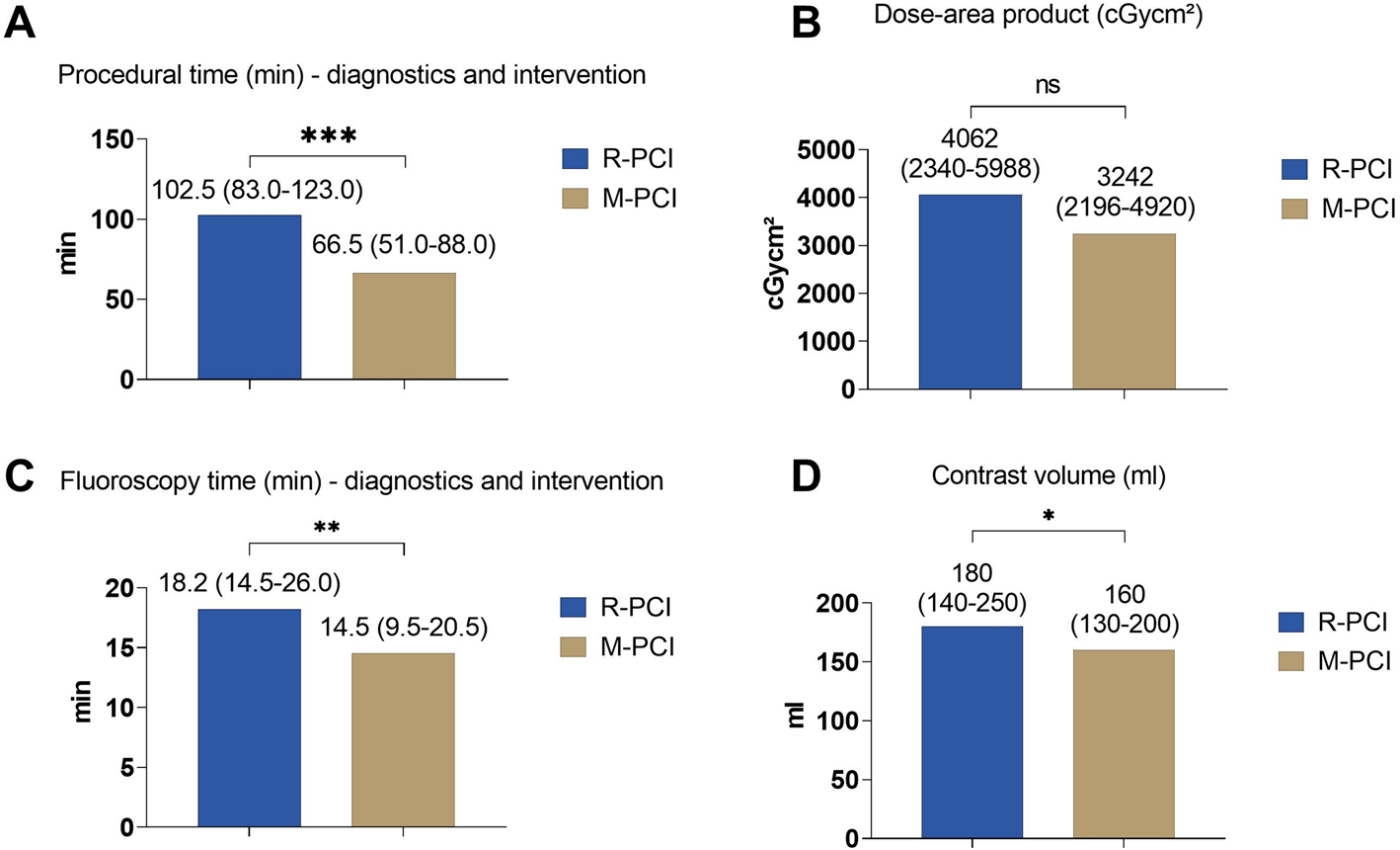
Comparison of procedural characteristics of Robotic assisted PCI (R-PCI, *n* = 70) with matched manual PCI cohort (M-PCI, *n* = 70). ns = *p* ≥ 0.05; * = *p* < 0.05; ** = *p* < 0.01; *** = *p* < 0.001. *PCI* percutaneous coronary intervention

The periprocedural outcomes highlighted several significant differences between the two intervention techniques. Both groups treated a similar number of lesions, deployed a similar number of stents and used radial access in more than 70% of patients (Table 2). There was no difference in the PCI success rate (70 (100.0%) vs. 66 (94.3%), *p* = 0.120) nor in MACE (0 (0.0%) vs.1 (1.4%), *p* = 1.000) between R-PCI and M-PCI. Notably, the intervention time was significantly higher in the R-PCI group compared to the M-PCI group (102.5 (83.0–123.0) min vs. 66.5 (51.0–88.0) min, *p* < 0.001, Fig. 1). Similarly, fluoroscopy time (18.2 min (14.5–26.0) vs. 14.5 (9.5–20.5) min, *p* = 0.002) and contrast volume used (180.0 (140–250) ml vs. 160.0 (130–200) ml, *p* = 0.041) were higher in the R-PCI group. Importantly, there was no difference in dose-area product (4061.9 (2340.0–5988.3) vs. 3241.7 (2196.1–4919.8) cGycm^2^, *p* = 0.361).

**Table 2:** Periprocedural outcome

	M-PCI (*n* = 70)	R-PCI (*n* = 70)	*p* value
Lesions treated (*n*)	1.0 (1.0–1.0)	1.0 (1.0–1.0)	0.877
Number of stents deployed (*n*)	1.0 (1.0–2.0)	1.0 (1.0–1.0)*	0.837
Radial access site (artery)	50 (71.4%)	51 (72.9%)	1.000
Successful PCI	66 (94.3%)	70 (100.0%)	0.120
Primary lesion—remaining stenosis (%)	0.0 (0.0–0.0)	0.0 (0.0–0.0)	0.747
MACE (major adverse cardiovascular events)	1 (1.4%)	0 (0.0%)	1.000
Myocardial infarction after PCI	0 (0.0%)	0 (0.0%)	
CK-MB elevation	0 (0.0%)	0 (0.0%)	
Dissection	1 (1.4%)	0 (0.0%)	1.000
Stent thrombosis	0 (0.0%)	0 (0.0%)	
Pericardial effusion	0 (0.0%)	0 (0.0%)	
Target lesion revascularization (TLR)	0 (0.0%)	0 (0.0%)	
Hospital length of stay (d)	3.0 (2.0–5.0)	3.0 (2.0–6.0)	0.772
ICU stay post PCI	16 (22.9%)	10 (14.3%)	0.277
Hospital survival	70 (100.0%)	70 (100.0%)	

*R-PCI: Mean number of stents deployed: 1.2 ± 0.6 SD

*CK-MB* creatine kinase myocardial band isoenzyme, *ICU* intensive care unit, *MACE* major adverse cardiovascular events, *M-PCI* manual PCI, *PCI* percutaneous coronary intervention, *R-PCI* robotic-assisted PCI

### Long-term follow-up

**Fig. 2:**
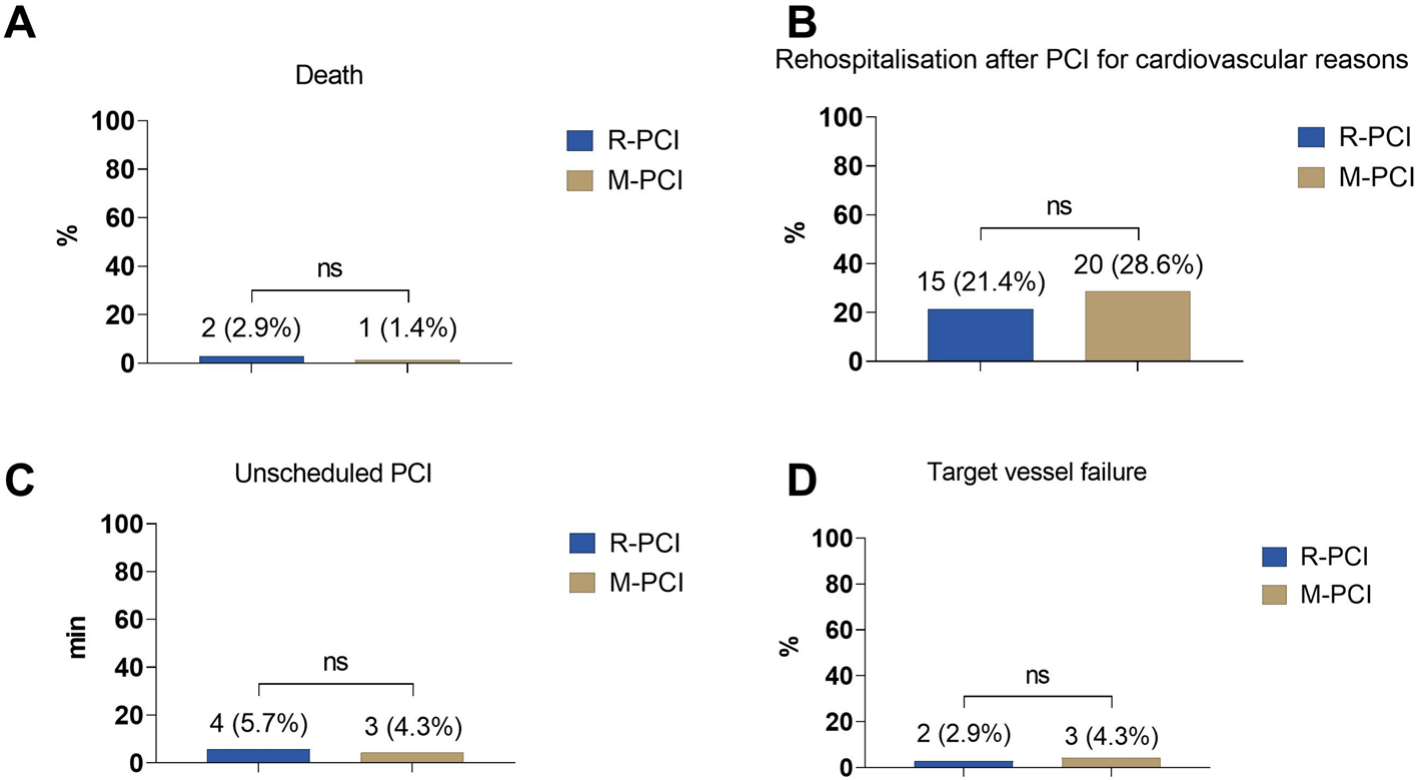
Comparison of one-year outcome of Robotic assisted PCI (R-PCI, *n* = 70) with matched manual PCI cohort (M-PCI, *n* = 70). ns = *p* ≥ 0.05; * = *p* < 0.05; ** = *p* < 0.01; *** = *p* < 0.001. *PCI* percutaneous coronary intervention

Successful 1 year follow-up for all R-PCI and M-PCI patients showed no difference in mortality between (2 (2.9%) vs. 1 (1.4%), *p* = 0.884, Fig. 2 and supplemental figure S2). The cumulative rate of rehospitalisation after PCI for cardiovascular reasons was similar in both groups (15 (21.4%) vs. 20 (28.6%), *p* = 0.560, Fig. 2) as well as the time to first rehospitalization (supplemental figure S3). Moreover, there was no difference regarding the need for unscheduled PCI (4 (5.7%) vs. 3 (4.3%), *p* = 0.671, Fig. 2 and supplemental figure S4) or target vessel failure (2 (2.9%) vs. 3 (4.3%), *p* = 0.564, Fig. 2). A second, focused propensity score matching analysis, in which the variables age and body mass index were also taken into account, revealed similar results for the 1-year outcome (supplemental table S4).

HRQL follow up showed similar results for NYHA and CCS score after 6 and 12 months (supplemental table S5 and S6). Apart from EQ-5D-5L usual activities after 6 months (1.5 (1.0–3.0) vs. 1.0 (1.0–1.5), *p* = 0.035), there were no further differences in the EQ-5D-5L and SAQ7 total score or their subcategories after 6 and 12 months (supplemental table S5 and S6).

## Discussion

This study evaluated the CorPath robotic system (CorPath GRX) in a highly standardized, prospective follow-up with a control group of manual PCI patients treated by the same team of interventionalists in the same period of time. In this matched pair analysis between 70 R-PCI and 70 M-PCI patients, this study showed a high success rate and solid safety profile for R-PCI. Of note, several periprocedural characteristics, such as procedural time, fluoroscopy time and contrast volume, were in favour of M-PCI, even though CTO and bifurcation PCIs were not excluded in this group but excluded from R-PCI. Against the background of this limitation, long-term results of R-PCI were favourable and 1-year outcomes were similar to M-PCI.

### Periprocedural outcome

In this study, there was no difference in the success rate between R-PCI and M-PCI, while patients had the same severity level of coronary lesion in both groups, according to the ACC classification and Syntax score. This confirms that R-PCI can be used successfully, with the limitation of manual support in some cases. Thereby, our study is in line with the results of Patel et al*.* and Bay et al. that also showed equal success rate in R-PCI and M-PCI [7, 19].

Importantly, there were also no differences in the MACE rate between the two groups. Thus, the results of this study confirm the high safety profile of previous studies [1–3, 10]. Only the study by Mahmud et al*.* reported a MACE rate after R-PCI of 7.5%. However, in this study the R-PCI MACE rate was comparable to the manual control group [4]. In our point of view, this is especially important as safety is a highly relevant aspect of this technology, as the interventionalist is completely deprived of tactile feedback with R-PCI and has to rely solely on visual feedback.

The rate of manual assistance for R-PCI was 19%. This value is of course highly dependent on the complexity of the lesions that are challenged by robotics. Based on the ACC classification and the Syntax score, a relevant part of the patients in this study had complex lesions with severe calcifications and tortuosity, which contribute to this manual assistance rate. Manual assistance rate in comparative studies was between 10 and 15% [3, 6] and the study by Mahmud et al*.* which focused specifically on complex coronary lesions also showed a manual conversion rate of 11% [4]. This illustrates that complex lesions remain a relevant limitation of this technology as in these cases the manual expertise of a very experienced internationalist is needed. Moreover, it challenges concepts of long-range remote control, as the experienced internationalist is still required on site.

In addition to the success rate and safety for the patient, there are other relevant aspects such as procedure duration, radiation exposure and contrast agent usage.

In this study, R-PCI required a median of 36 min more time than M-PCI. This difference is mainly due to the alignment and preparation of the robotic arm. Nevertheless, fluoroscopy time also showed a significantly longer duration of approx. 4 min for R-PCI. This could be interpreted as an indicator that performing the PCI using the control console without haptic feedback was slower than with conventional coronary intervention.

R-PCI also required a median of 20ml more contrast agent than M-PCI. Since the total contrast medium consumption of 180 ml for a combined diagnostic and therapeutic PCI is still below the reference limit of 250 ml, a clinical disadvantage in this respect is questionable [20]. In contrast to the results of this study, however, Patel et al*.* and Mahmud et al*.* were able to show that there were no differences between R-PCI and M-PCI in terms of fluoroscopy time and contrast agent requirement [4, 7]. Furthermore, there was no significant difference between the groups in terms of radiation exposure for the patient (dose-area product) and exposure of the assistant in the catheter laboratory was 38 times lower than manual PCI [6]. The results of this study thus emphasize the radiation protection aspect of R-PCI.

### Long-term outcome

R-PCI showed favorable results in the long-term follow-up over 1 year. There were no differences in all-cause mortality compared to M-PCI or in the rate of unscheduled PCI within 12 months after the index PCI. In addition, as an important quality indicator, both cohorts showed a very low and comparable rate of target vessel failure. In a study comparing the one-year outcome of R-PCI patients with a retrospective manual cohort, Bay et al*.* showed similar results with a low and equal rate of TVR between both treatment techniques [19]. The study by Walters et al*.* reached the same conclusion with a similar TVR rate of 1% vs. 1.9% for R-PCI and M-PCI after 12 months in patients with complex coronary lesions. [5]

Moreover, in this study, there were no differences between both treatment groups in terms of HRQL with regard to NYHA and CCS scores, SAQ7 and EQ-5D-5L at both 6 and 12 months after index PCI. In line with these results, the rate of rehospitalisation was also comparable between the groups. In summary, robotic PCI shows favourable and comparable results to conventional PCI in long-term follow-up, not only with regard to the interventional success but also with regard to morbidity.

### Limitations

This is an observational study without a randomised design and therefore contains the risk of selection bias. Since the patients were screened and selected by the treating interventionalists based on fundamental feasibility of R-PCI, and CTO, bifurcation lesions as well as STE-ACS patients were excluded from R-PCI, there is a certain selection bias in these patients. Nevertheless, multiple methodical measures were taken to reduce this bias, such as prospective study design with prospective follow-up and predefined endpoints, establishment of a control cohort which was treated by the same team of interventionalists during the same time period. In addition, a matched pair analysis was carried out on the severity and complexity of the coronary lesions to be treated, to further reduce selection bias. Moreover, as this is a single centre study, different workflows and standards at other centres may limit the comparability of this study.

## Conclusion

Robotics in interventional cardiology is a promising technology with great potential. The results of this study and other publications indicate that the procedure is not inferior to manual PCI with regard to less complex lesions. However, there are relevant limitations, particularly in the area of complex coronary lesions. With consequent further development of the current technology, robotics could open up valuable treatment options in future.

## Supplementary Information

Below is the link to the electronic supplementary material.

Supplementary file1 (DOCX 572 KB)

## Data Availability

The datasets used and/or analysed during the current study are available from the corresponding author on reasonable request, after deidentification, to achieve aims in the approved proposal. Proposals should be directed to jonathan.rilinger@universitaets-herzzentrum.de.

## Abbreviations

ACC/AHA: American College of Cardiology/Amercian Heart Association
BMI: Body mass index
CABG: Coronary artery bypass grafting
CAD: Coronary artery disease
CCS: Canadian Cardiovascular Society
CK: Creatine kinase
CK-MB: Creatine kinase myocardial band isoenzyme
EQ-5D-5L: EuroQol-5-Dimensions-5-Levels questionnaire
HRQL: Health related quality of life
ICU: Intensive care unit
iFR: Instantaneous wave-free ratio
LAD: Left anterior descending coronary artery
LCX: Left circumflex coronary artery
LM: Left main coronary artery
LVEF: Left ventricular ejection fraction
MACE: Major adverse cardiovascular events
MI: Myocardial infarction
M-PCI: Manual PCI
NSTE-ACS: Non-ST-segment elevation acute coronary syndrome
NYHA: New York Heart Association
OCT: Optical coherence tomography
PCI: Percutaneous coronary intervention
R-PCI: Robotic-assisted PCI
RCA: Right coronary artery
SAQ-7: Seattle Angina Questionnaire (7 items)
STE-ACS: ST-segment elevation acute coronary syndrome
SYNTAX: Synergy between PCI with taxus and cardiac surgery
TLR: Target lesion revascularization
