# Supplementary material for: One-year outcome of robotical vs. manual percutaneous coronary intervention
Source: Clin Res Cardiol. 2024 Aug 21;114(8):1000–7. doi: 10.1007/s00392-024-02524-0 (PMC12283869; doi:10.1007/s00392-024-02524-0)
Supplement: Supplementary file 1 — Supplementary file1 (DOCX 572 KB) [file 392_2024_2524_MOESM1_ESM.docx]

# **Online data supplement – One-year outcome of robotical vs. manual percutaneous coronary intervention**

Constantin von zur Mühlen, Marvin Jeuck, Timo Heidt, Thomas Maulhardt, Tau Hartikainen, Alexander Supady, Ingo Hilgendorf, Dennis Wolf, Klaus Kaier, Dirk Westermann, Jonathan Rilinger

#

Continuous variables are presented as median (25th-75th percentile) and the Mann-Whitney test is applied. For categorical variables, N (%) are shown and Fisher's exact test is applied.

## Figures


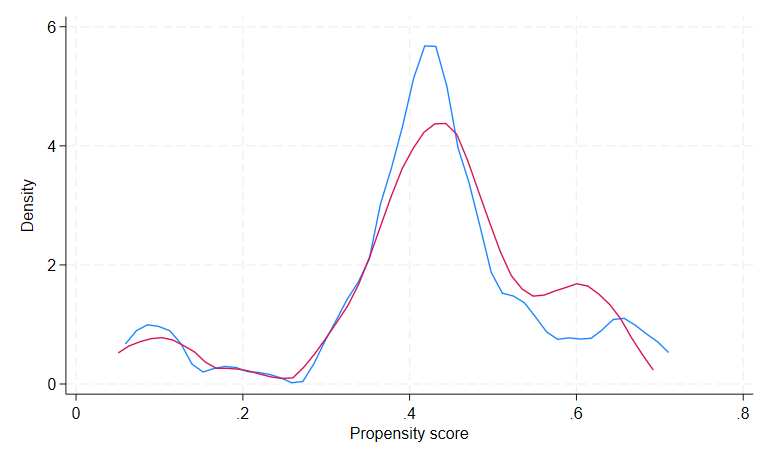


### **Figure S1. Overlap plot for matched cohort**

The red line represents patients undergoing (R-PCI) Robotic PCI and the blue line represents patients with no Robotic PCI (M-PCI).


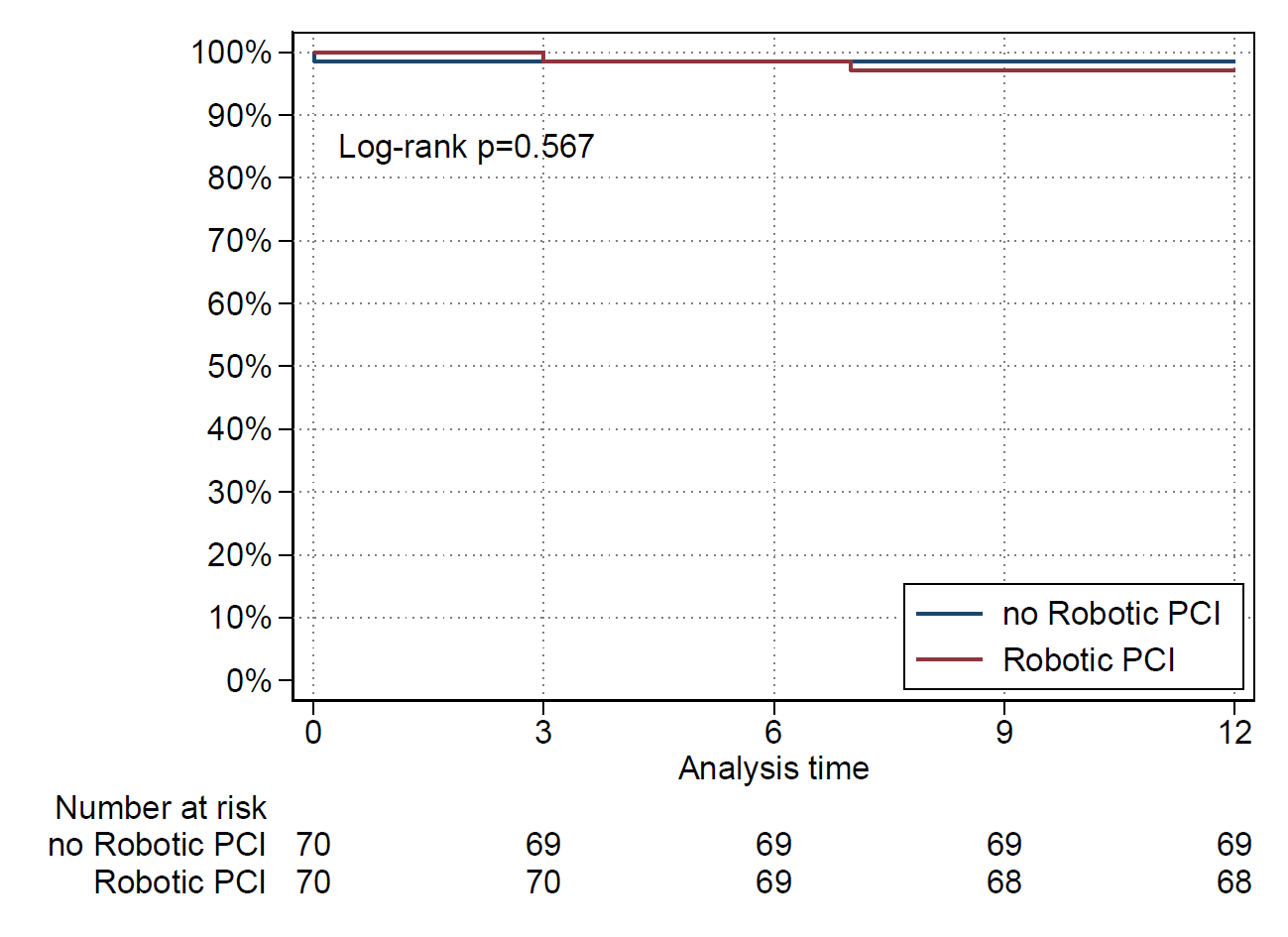


### **Figure S2. Time to death analysis**

*PCI: percutaneous coronary intervention.*


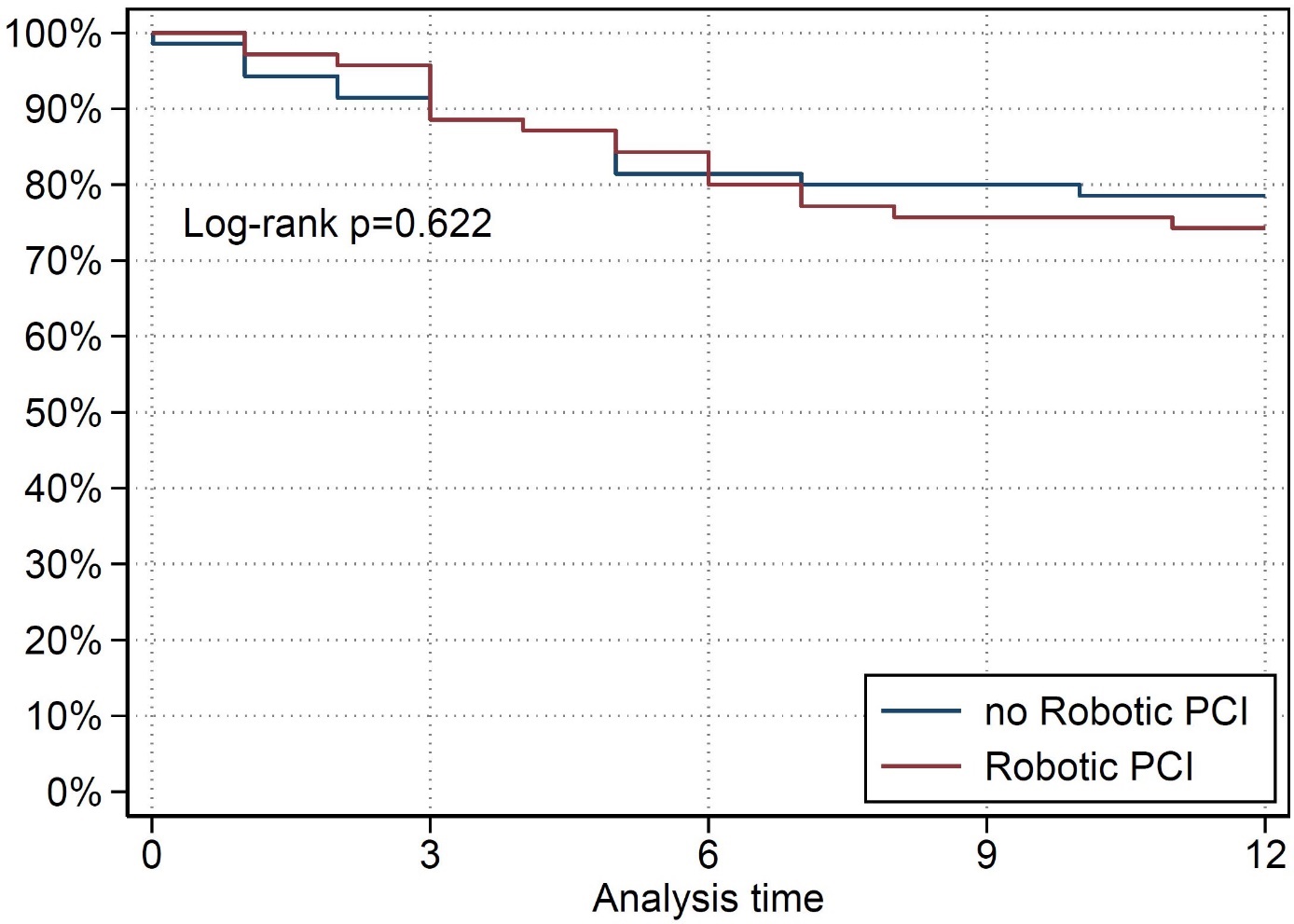


### **Figure S3. Time to first rehospitalization**

*PCI: percutaneous coronary intervention.*


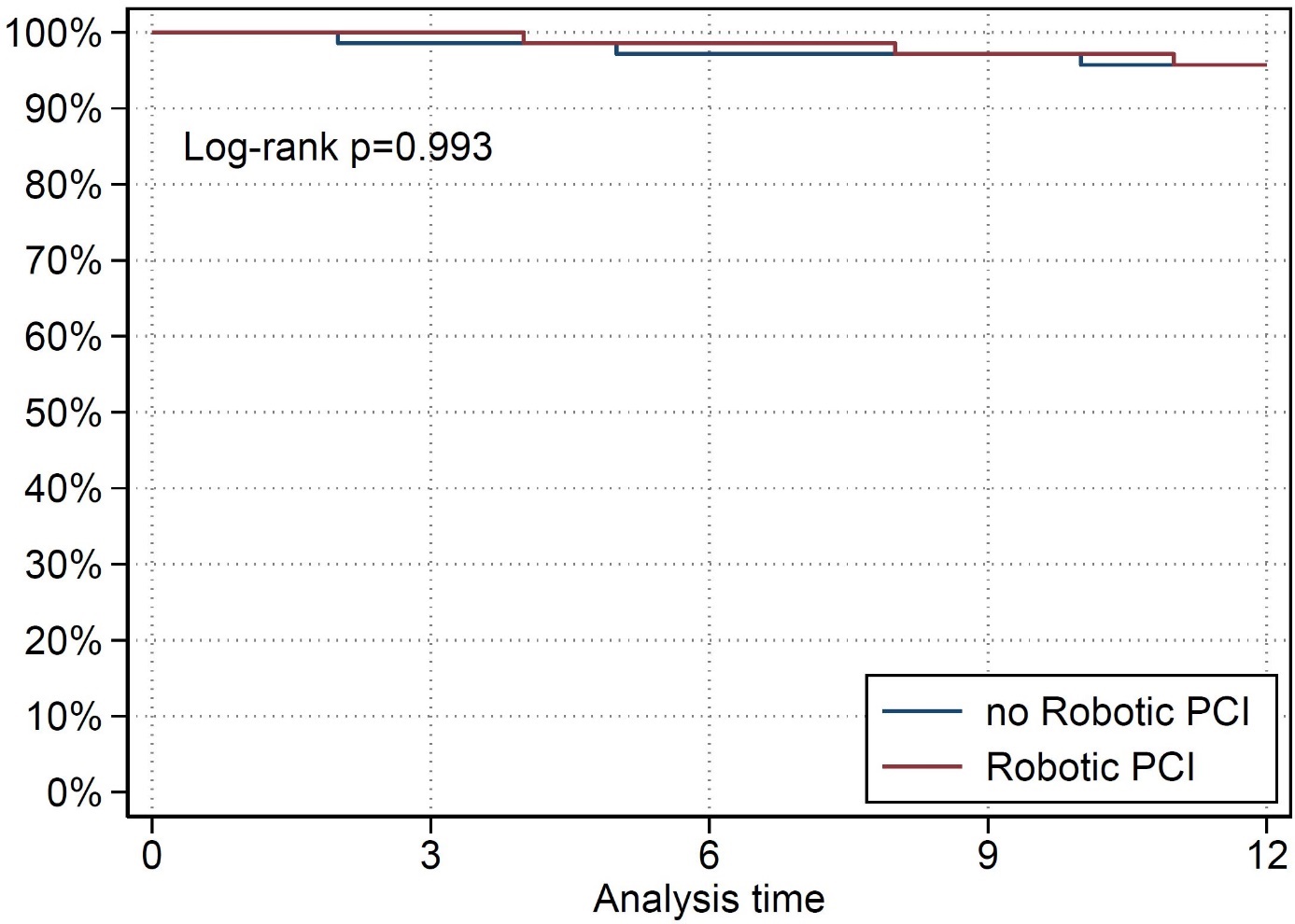


### **Figure S4. Time to first unscheduled PCI**

*PCI: percutaneous coronary intervention.*

## **Tables**

### **Table S1. Procedural steps of manual PCI (M-PCI) vs. robotic PCI (R-PCI)**

|  | M-PCI | R-PCI |
| --- | --- | --- |
| Radial or femoral sheath application | manual | manual |
| Guide catheter insertion | manual | manual |
| Loading the sterile cassette of the robotic arm with coronary wires and balloon/stent catheters | - | manual |
| Control of the coronary wire and passage of the lesion | manual | robotically |
| Advancing of the balloon/stent catheter | manual | robotically |
| Dilatation of the balloon/stent catheter | manual | manual |
| Removement of the coronary wires and balloon/stent catheters | manual | robotically |
| Guide catheter removement | manual | manual |

### **Table S2. Advanced baseline characteristics of matched cohort**

|  | M-PCI  (n=70) | R-PCI  (n=70) | P value |
| --- | --- | --- | --- |
| **Pre-existing conditions** |  |  |  |
| Peripheral vascular disease | 7 (10.0%) | 5 (7.1%) | 0.764 |
| Stroke or TIA | 7 (10.0%) | 14 (20.0%) | 0.154 |
| Chronic dialysis | 1 (1.4%) | 1 (1.4%) | 1.000 |
| Liver cirrhosis | 0 (0.0%) | 1 (1.4%) | 1.000 |
| Pulmonary diseases | 8 (11.4%) | 11 (15.7%) | 0.623 |
| **Laboratory pre PCI** |  |  |  |
| Hemoglobin (g/dl) | 13.8 (12.4-14.8) | 14.0 (12.9-14.9) | 0.754 |
| Creatinine PCI (mg/dl) | 1.1 (0.9-1.2) | 1.0 (0.9-1.1) | 0.078 |
| CK (U/l) | 98.0 (71.0-137.0) | 135.0 (87.0-184.0) | **0.021** |
| CK-MB (U/l) | 36.0 (19.0-43.0) | 27.0 (18.0-44.0) | 0.645 |
| Myoglobin (ng/ml) | 86.0 (60.0-106.0) | 80.0 (51.5-108.5) | 0.558 |
| Troponin ng/ml | 31.4 (22.3-91.0) | 98.8 (13.1-307.0) | 0.371 |
| proBNP pre PCI (pg/ml) | 655.0 (198.0-1257.0) | 410.5 (138.5-811.5) | 0.163 |
| **Laboratory post PCI** |  |  |  |
| Creatinine PCI (mg/dl) | 1.1 (1.0-1.4) | 1.1 (1.0-1.3) | 0.346 |
| CK (U/l) | 151.0 (66.0-333.0) | 124.5 (83.0-209.5) | 0.397 |
| CK-MB (U/l) | 27.5 (19.5-68.5) | 24.5 (13.0-34.0) | 0.512 |
| Myoglobin (ng/ml) | 91.5 (67.0-136.0) | 81.0 (49.0-107.0) | 0.208 |
| Troponin ng/ml | 213.0 (32.2-294.0) | 20.5 (10.4-148.0) | 0.297 |

*BNP: brain natriuretic peptide; CK: creatine kinase; CK-MB: creatine kinase myocardial band isoenzyme; M-PCI: manual PCI; PCI: percutaneous coronary intervention; R-PCI: robotic-assisted PCI.*

### **Table S3. Advanced periprocedural characteristics of matched cohort**

|  | M-PCI  (n=70) | R-PCI  (n=70) | P value |
| --- | --- | --- | --- |
| Extend of coronary artery disease |  |  |  |
| Single-vessel disease | 11 (15.7%) | 15 (21.4%) | 0.085 |
| Three-vessel disease | 43 (61.4%) | 30 (42.9%) |  |
| Two-vessel disease | 16 (22.9%) | 25 (35.7%) |  |
| Location of primary lesion |  |  |  |
| LM (left main coronary artery) | 2 (2.9%) | 1 (1.4%) | 1.000 |
| LAD (left anterior descending coronary artery) | 29 (41.4%) | 32 (45.7%) | 0.733 |
| LCX (left circumflex coronary artery) | 21 (30.0%) | 16 (22.9%) | 0.444 |
| RCA (right coronary artery) | 18 (25.7%) | 21 (30.0%) | 0.706 |
| Bifurcation lesion treated | 8 (11.4%) | 0 (0.0%) | **0.006** |
| Chronic total occlusions treated | 4 (5.7%) | 0 (0.0%) | 0.120 |
| Primary lesion stenosis (%) | 90.0 (80.0-99.0) | 90.0 (80.0-90.0) | **0.048** |
| Treated vessel In-stent-restenosis | 7 (10.0%) | 9 (12.9%) | 0.791 |
| Treated aorto-ostial lesion | 6 (8.6%) | 4 (5.7%) | 0.745 |
| Severe Tortuosity | 6 (8.6%) | 10 (14.3%) | 0.426 |
| Thrombus | 2 (2.9%) | 0 (0.0%) | 0.496 |
| Appliciation of ≥ 2 coronary wires | 34 (48.6%) | 12 (17.1%) | **<0.001** |
| Advanced coronary diagnostics and therapy |  |  |  |
| iFR - SyncVision use | 9 (12.9%) | 17 (24.3%) | 0.127 |
| OCT use | 4 (5.7%) | 10 (14.3%) | 0.157 |
| IVL use | 3 (4.3%) | 2 (2.9%) | 1.000 |
| Rotablator use | 1 (1.4%) | 0 (0.0%) | 1.000 |

*iFR: instantaneous wave-free ratio; M-PCI: manual PCI; OCT: optical coherence tomography; PCI: percutaneous coronary intervention; R-PCI: robotic-assisted PCI.*

### **Table S4. One-year follow up with advanced* propensity score matching**

|  | M-PCI  (n=70) | R-PCI  (n=70) | P value |
| --- | --- | --- | --- |
| Death | 1 (1.4%) | 2 (2.9%) | 0.559 |
| Rehospitalisation after PCI for cardiovascular reasons | 18 (25.7%) | 15 (21.4%) | 0.704 |
| Unscheduled PCI | 4 (5.7%) | 4 (5.7%) | 1.000 |
| Target vessel failure | 3 (4.3%) | 2 (2.9%) | 0.649 |

*advanced Propensity score matching: Matching for “reason for PCI, Modified ACC/AHA Lesion-Specific Classification of primary lesion and SYNTAX I score” as well as age and body mass index.

*PCI: percutaneous coronary intervention; R-PCI: robotic-assisted PCI.*

### **Table S5. HRQL** **(health related quality of life) follow-up at 6 months of matched cohort**

|  | M-PCI  (n=70) | R-PCI  (n=70) | P value |
| --- | --- | --- | --- |
| NYHA score | 1.0 (1.0-2.0) | 1.5 (1.0-2.0) | 0.179 |
| CCS angina scale | 0.0 (0.0-0.0) | 0.0 (0.0-0.0) | 0.336 |
| **EQ-5D-5L** |  |  |  |
| Mobility | 1.0 (1.0-2.0) | 1.0 (1.0-2.0) | 0.582 |
| Self-care | 1.0 (1.0-1.0) | 1.0 (1.0-1.0) | 0.251 |
| Usual activities | 1.0 (1.0-1.5) | 1.5 (1.0-3.0) | **0.035** |
| Pain/Discomfort | 2.0 (1.5-3.0) | 2.5 (1.0-3.0) | 0.334 |
| Anxiety/Depression | 1.0 (1.0-2.0) | 1.0 (1.0-2.0) | 0.618 |
| Total score | 0.9 (0.8-0.9) | 0.9 (0.6-0.9) | 0.185 |
| **SAQ7** |  |  |  |
| Physical Limitation | 100.0 (100.0-100.0) | 100.0 (100.0-100.0) | 0.605 |
| Angina Frequency | 100.0 (100.0-100.0) | 100.0 (90.0-100.0) | 0.309 |
| Quality of Life | 100.0 (87.5-100.0) | 100.0 (87.5-100.0) | 0.421 |
| Summary score | 100.0 (95.8-100.0) | 100.0 (89.2-100.0) | 0.375 |

*CCS: Canadian Cardiovascular Society; EQ-5D-5L: EuroQol-5-Dimensions-5-Levels questionnaire; M-PCI: manual PCI; NYHA: New York Heart Association; PCI: percutaneous coronary intervention; R-PCI: robotic-assisted PCI; SAQ-7: Seattle Angina Questionnaire (7 items).*

### **Table S6. HRQL follow-up at 12 months of matched cohort**

|  | M-PCI  (n=70) | R-PCI  (n=70) | P value |
| --- | --- | --- | --- |
| NYHA score | 1.0 (1.0-2.0) | 1.0 (1.0-2.0) | 0.291 |
| CCS angina scale | 0.0 (0.0-0.0) | 0.0 (0.0-0.0) | 0.519 |
| **EQ-5D-5L** |  |  |  |
| Mobility | 1.0 (1.0-2.0) | 1.0 (1.0-3.0) | 0.511 |
| Self-care | 1.0 (1.0-1.0) | 1.0 (1.0-1.0) | 0.860 |
| Usual activities | 1.0 (1.0-3.0) | 1.0 (1.0-3.0) | 0.887 |
| Pain/Discomfort | 2.0 (1.0-3.0) | 2.0 (1.0-3.5) | 0.481 |
| Anxiety/Depression | 1.0 (1.0-1.5) | 1.0 (1.0-1.5) | 0.937 |
| Total score | 0.9 (0.8-1.0) | 0.9 (0.7-1.0) | 0.737 |
| **SAQ7** |  |  |  |
| Physical Limitation | 100.0 (100.0-100.0) | 100.0 (100.0-100.0) | 0.689 |
| Angina Frequency | 100.0 (100.0-100.0) | 100.0 (100.0-100.0) | 0.431 |
| Quality of Life | 100.0 (100.0-100.0) | 100.0 (81.2-100.0) | 0.267 |
| Summary score | 100.0 (100.0-100.0) | 100.0 (92.1-100.0) | 0.274 |

*CCS: Canadian Cardiovascular Society; EQ-5D-5L: EuroQol-5-Dimensions-5-Levels questionnaire; M-PCI: manual PCI; NYHA: New York Heart Association; PCI: percutaneous coronary intervention; R-PCI: robotic-assisted PCI; SAQ-7: Seattle Angina Questionnaire (7 items).*
